# Strain Distribution in Root Surface Dentin of Maxillary Central Incisors during Lateral Compaction

**DOI:** 10.1371/journal.pone.0156461

**Published:** 2016-05-26

**Authors:** Raphael Pilo, Zvi Metzger, Tamar Brosh

**Affiliations:** 1 Departments of Oral Rehabilitation, Goldschleger School of Dental Medicine, Tel-Aviv University, Tel-Aviv, Israel; 2 Departments of Endodontology, Goldschleger School of Dental Medicine, Tel-Aviv University, Tel-Aviv, Israel; 3 Departments of Oral Biology, Goldschleger School of Dental Medicine, Tel-Aviv University, Tel-Aviv, Israel; University of Brescia, ITALY

## Abstract

**Aim:**

To precisely quantify the circumferential strains created along the radicular dentin of maxillary incisors during a simulated clinical procedure of lateral compaction.

**Methods:**

Six miniature strain gauges were bonded on the roots of fourteen recently extracted maxillary central incisors that were subjected to root canal instrumentation. The strain gauges were bonded at three levels (apical, middle, and coronal) and four aspects (buccal, lingual, mesial, and distal) of the roots. Each tooth was embedded in a PVC cylinder containing polyvinyl-siloxane impression material. Root filling was then performed by simulating the clinical procedure of lateral compaction using nickel-titanium finger spreaders. The force applied to the spreader and the strains developing in the surface root dentin were continuously recorded at a frequency of 10 Hz.

**Results:**

The highest strains that developed during lateral compaction were in the mesial and distal aspects at the apical level of the root. The magnitudes of the maximal mesial/distal strains at the apical as well as the mid-root levels were approximately 2.5–3 times higher than those at the buccal/lingual aspects (p = 0.041). The strains decreased significantly (p<0.04) from the apical through the mid-root levels to the coronal level, yielding gradients of 2.5- and 6-fold, respectively. The mesial and distal strains were consistently tensile and did not differ significantly; however, the buccal strains were generally 35–65% higher than the lingual strains (p = 0.078). Lateral compaction resulted in the gradual build-up of residual strains, resulting in generation of a 'stair-step' curve. These strains declined gradually and almost completely disappeared after 1000 sec.

**Conclusions:**

With proper mounting of several miniature strain gauges at various levels and aspects of the root, significant circumferential strains can be monitored under clinically relevant compaction forces. The residual strains at the end of lateral compaction are not stored in the dentin but decrease gradually to negligible levels.

## Introduction

Lateral compaction, frequently cited as the 'gold standard' technique for root canal filling, is one of the most commonly used methods and is utilized by more than half of all practitioners worldwide [1–3]. Traditionally, lateral compaction was applied using hand spreaders [4], but it is currently applied mainly using finger spreaders. Such spreaders exert less lateral force on the canal walls and allow for greater tactile sense [5]. Nickel-titanium (NiTi) finger spreaders are preferred over the less elastic stainless steel ones [5]. They generate less stress during lateral compaction [6], have better penetration capabilities [7–9] and result in decreased apical microleakage in endodontically treated teeth [10].

The apical force applied through finger spreaders during compaction of gutta-percha, which is currently the most commonly used root filling material, exerts pressure on the material, resulting in circumferential tensile stresses on the canal surface [11]. Such stresses are a concern, especially when preexisting micro-cracks are present in the dentin, with potential propagation from a subcritical to a critical length [12]. These cracks may subsequently develop into vertical root fracture (VRF). Such micro-cracks in radicular dentin might be created by rotary NiTi instruments through the process of canal instrumentation [13, 14].

Because a relationship between lateral compaction and root fracture has been proposed [11, 15–17], the characterization of strains created in radicular dentin during lateral compaction has been a research area of much interest. Their magnitude is important because crack initiation and propagation are mainly controlled by tensile strain [16, 18]. Such localized strains have been previously studied using fracture mechanics analysis [11], finite element analysis (FEA) [19–24], photoelastic models [25, 26] and strain gauges [4, 15, 27–29].

Previous studies using strain gauges have revealed a lack of differences between apical and mid-root strains (as measured on the buccal surface) and higher strain generation resulting from hand spreaders compared with stainless steel (SS) finger spreaders. The limitations of these prior studies involve the use of strain gauges with large dimensions, with lengths of both 3.18–5.84 mm [4, 15, 28, 30] and 1.57 mm [27]. Gauges with large dimensions measure the average strain over that area and may thus obscure strain peaks that could be detected over a much smaller area; the reported, insignificant strain readings have been below 50 micro strains (μS) [4, 15, 27–28, 30]. In all of these studies, only the buccal surface was chosen as the strain gauge mounting site because clinically, the fracture plane is typically buccal-lingual [31–33]. In a more recent study, four miniature strain gauges (0.79–1.57 mm gauge length) were bonded between the apical and middle third of maxillary and mandibular incisor roots on the buccal, lingual, mesial and distal surfaces; they were subsequently loaded by a hand spreader via a loading machine until fracture [29]. Such loading does not simulate the clinical procedure of lateral compaction, which is typically repetitive in nature. The surface strains that were monitored on maxillary incisors roots were consistently tensile on the proximal surfaces (607–2893 μS) and were much lower (either compressive or tensile) on the buccal-lingual surfaces (-324 to +254 μS). The same trend has been reported for mandibular incisor roots, with values of 462–11608 μS and -3894 to -199 μS for the mesial-distal and bucco-lingual surfaces, respectively [29]. However, these surface strains were obtained using a destructive model, with applied loads that reached, at fracture, levels of ~120–215 N and ~62–88 N for the maxillary and mandibular incisors, respectively. These loads greatly exceed the 15- to 30-N range of force that is used in daily clinical practice during the process of root canal obturation [34–35]. Moreover, no attempts have been made to monitor the strains along the roots. Such attempts were performed using FEA [19–20] or photoelastic models [25], showing that the highest strains that developed in root dentin during lateral compaction were located around the tip of the spreader, especially in the apical area. However, real teeth were not used in these models; thus, they only approximate the mechanical properties of dentin and the morphologies of teeth. Furthermore, the clinical procedure was not adequately simulated in these studies.

The aim of this study was to quantify the local circumferential strains created in the radicular dentin of maxillary incisors during a clinically simulated procedure of lateral compaction. The working hypothesis was that with proper mounting of multiple miniature strain gauges, accurate registration and differentiation of the circumferential strains at the various levels and aspects of the roots could be obtained under clinically relevant compaction forces and proper simulation of the clinical procedure.

## Materials and Methods

Single-rooted, intact maxillary central incisors that were extracted for periodontal reasons were collected. The study protocols were approved by the Tel-Aviv University Ethics Committee, and each patient signed an informed consent form. After cleaning, the teeth were stored in saline containing 0.01% thymol at 5°C

Radiographs were used to verify the presence of a single patent root canal. Teeth were checked for pre-existing cracks in their roots using transmitted light (Sendolight- Sjödings Sendoline, Kista, Sweden) and a stereo-microscope at x20 magnification [36]; those that were found to have cracks were omitted from the study. Sixteen incisors that fulfilled all of the inclusion criteria were included in the current study and used within two weeks of extraction.

Endodontic access cavities were prepared, and canals were negotiated with #15K files, establishing a working length at 1 mm short of the apical foramen. Root canals were prepared using ProFile files (Dentsply-Maillefer, Ballaigues, Switzerland) up to size 35/.04, which were operated at 300 rpm to working length, and the apical 2mm was then further prepared with K files (Zipperer, Dentsply-DeTrey) up to size 50. The resulting preparation had a .04 taper, excluding the apical 2 mm which had a .02 taper. RC-Prep (Premier, Philadelphia, PA, USA) was used with each instrument, and irrigation with 1 ml of 3% sodium hypochlorite was performed after the use of each instrument. The teeth were maintained at 100% humidity until experimentation. The moisture of the inner side of the root was maintained by the continuous presence of saline in the canal.

### Strain gauges

**Fig 1:**
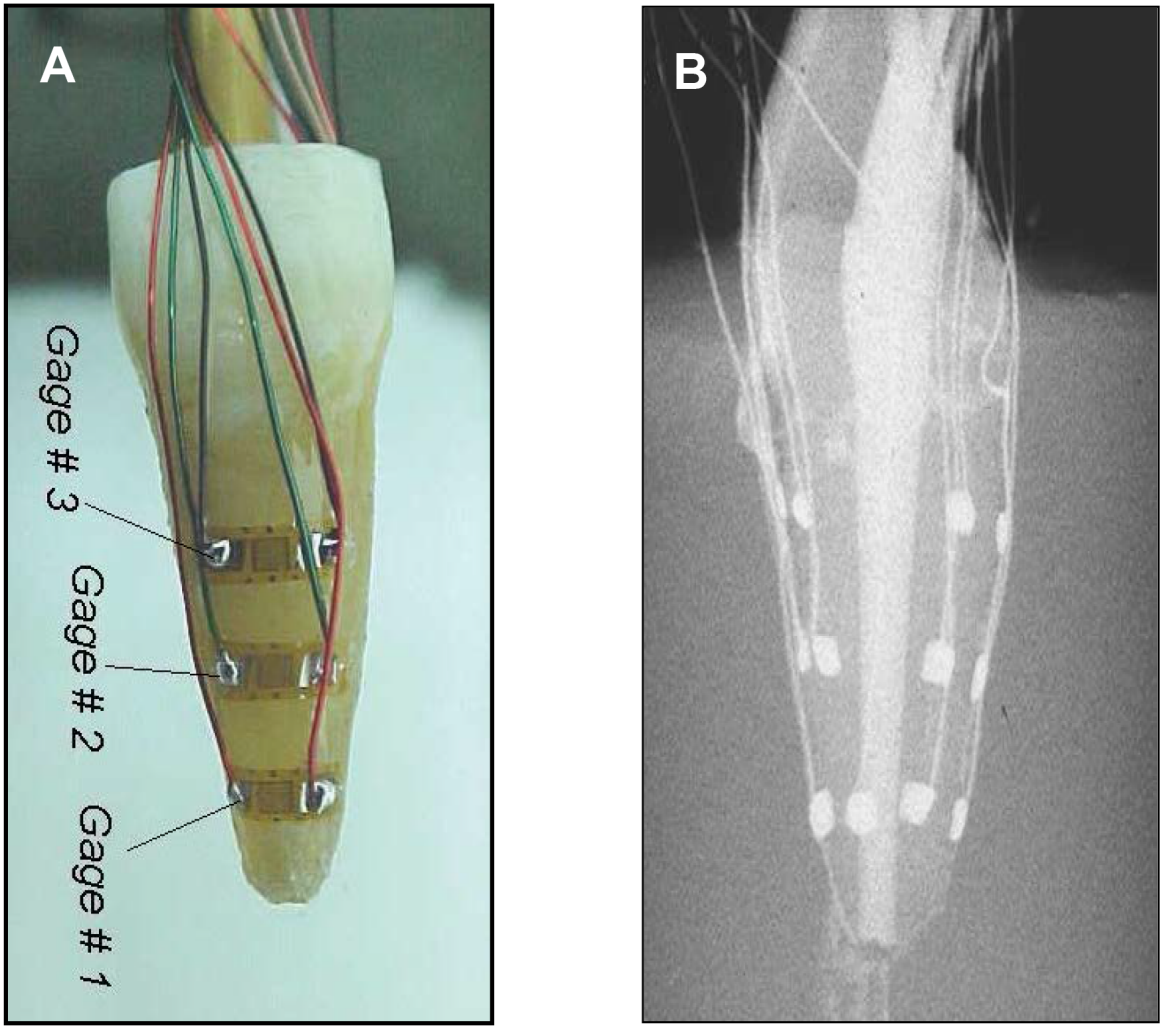
The experimental setup. (A) Maxillary central incisor with 6 miniature strain gauges mounted and wired according to configuration A. Three buccal gauges are shown (the three lingual gauges are not shown in the image). (B) Radiograph of the maxillary central incisor at the end of the lateral compaction procedure demonstrating the 6 mounted and wired strain gauges (mesial-distal view).

Six miniature strain gauges (EA-06-031DE-350 or EA-06-031EC-350, Micro Measurement Group, Raleigh, NC) with an active gauge length of 0.79 mm were bonded to the surface of each root. The roots were divided into coronal, middle and apical thirds, measuring from the CEJ to the apex, and the strain gauges were bonded in three levels at the middle of the root thirds. Seven configurations, with two teeth per configuration, were applied as follows (B = buccal, L = lingual, M = mesial, and D = distal):

1. apical (B+L), middle (B+L), coronal (B+L) (Fig 1a and 1b)
2. apical (M+D), middle (M+D), coronal (M+D)
3. apical (B+L+M+D), middle (B+L)
4. apical (B+L+M+D), middle (M+D)
5. apical (B+L+M+D), coronal (M+D)
6. middle (B+L+M+D), coronal (M+D)
7. middle (B+L+M+D), coronal (B+L)

In each configuration, the strain gauges were mounted to measure circumferential strains.

To compare the results obtained with the miniature strain gauges to those previously published using large gauges, a larger EA-06-125BT-120 strain gauge with an active gauge length of 3.18 mm was bonded to the buccal apical third of the roots of two additional incisor control teeth. These larger strain gauges were identical to those used by Dang and Walton [4] and by Lertchirakarn et al [15].

After completion of the lateral compaction procedure in these two control teeth, the strain gauges were carefully removed, and 6 miniature strain gauges were mounted on each tooth according to configuration D. The lateral compaction procedure was repeated.

### Strain gauge bonding

The strain gauge mounting protocol used in the present study was developed with and performed by the Department of Experimental Engineering, Vishay Israel, Netanya, Israel. The surface of the root was polished, and a thin layer of special epoxy cement was applied (M-Bond AE-10 Micro Measurements Group). Special cyanoacrylate cement (M-Bond 200, Micro Measurements Group) was used to bond the gauges to the root surface. The whole process was conducted under an operating microscope at x10 magnification. The humidity of the inner side of the root was maintained throughout the mounting procedure by the continuous presence of saline in the canal. The strain gauges were wired and connected to the measuring setup in a three-wire quarter Wheatstone bridge circuit design. They were then connected to a computerized data acquisition system (System 5000, Micro Measurement Group).

### Strain and force measurement setup and data collection

Each tooth was embedded in a PVC cylinder (3 cm diameter x 4.5 cm height) containing polyvinyl-siloxane impression material (Provil Novo M, Heraeus Kulzer, Wehrheim, Germany) to the level of the cementoenamel junction (CEJ), with the connecting wires extending coronally through the impression material.

The cylinder containing the tooth was placed on a horizontal plate equipped with a weighing load cell (Tedea, Netanya, Israel), which was wired to the same data acquisition system. Strain and force channels were calibrated between successive experiments.

A total of 7 data arrays were continuously acquired and registered as plots during the process of lateral compaction, including 6 strain plots and one force plot, all of which were obtained with a measurement frequency of 10 Hz. The plots appeared on a computer screen and were not observed by the operator who performed the lateral compaction; they were then stored as digital files for future reference and analysis.

### Root filling procedure

Lateral compaction was performed with the goal of simulating the clinical process as closely as possible. The root canal was filled with AH-26 (Dentsply-DeTrey, Konstanz, Germany) using a lentulo spiral filler and the master cone inserted to working length. Lateral compaction was applied using a NiTi finger spreader (NiTi B finger spreader, Dentsply-Maillefer, Ballaigue, Switzerland). For each spreader insertion, the length of entry, from a reference point at the incisal edge, was measured and recorded. Accessory cones (Dentsply-DeTrey) were added until the canal was full and no further spreader insertion was possible. The clinical use of spreaders was mimicked by applying a back-and-forth quarter circle rotating movement when the spreader was pushed inside of the canal. The spreader was always inserted along the palatal wall of the canal, with the force applied parallel to the long axis of the root. All lateral compaction procedures were performed by a single operator who was an experienced endodontist.

### Statistical analysis

Comparison of the mean maximal strains (as measured or normalized by the force applied at the maximal strains) at different locations along the roots was conducted using the paired t-test with a level of significance of 0.05.

## Results

**Fig 2:**
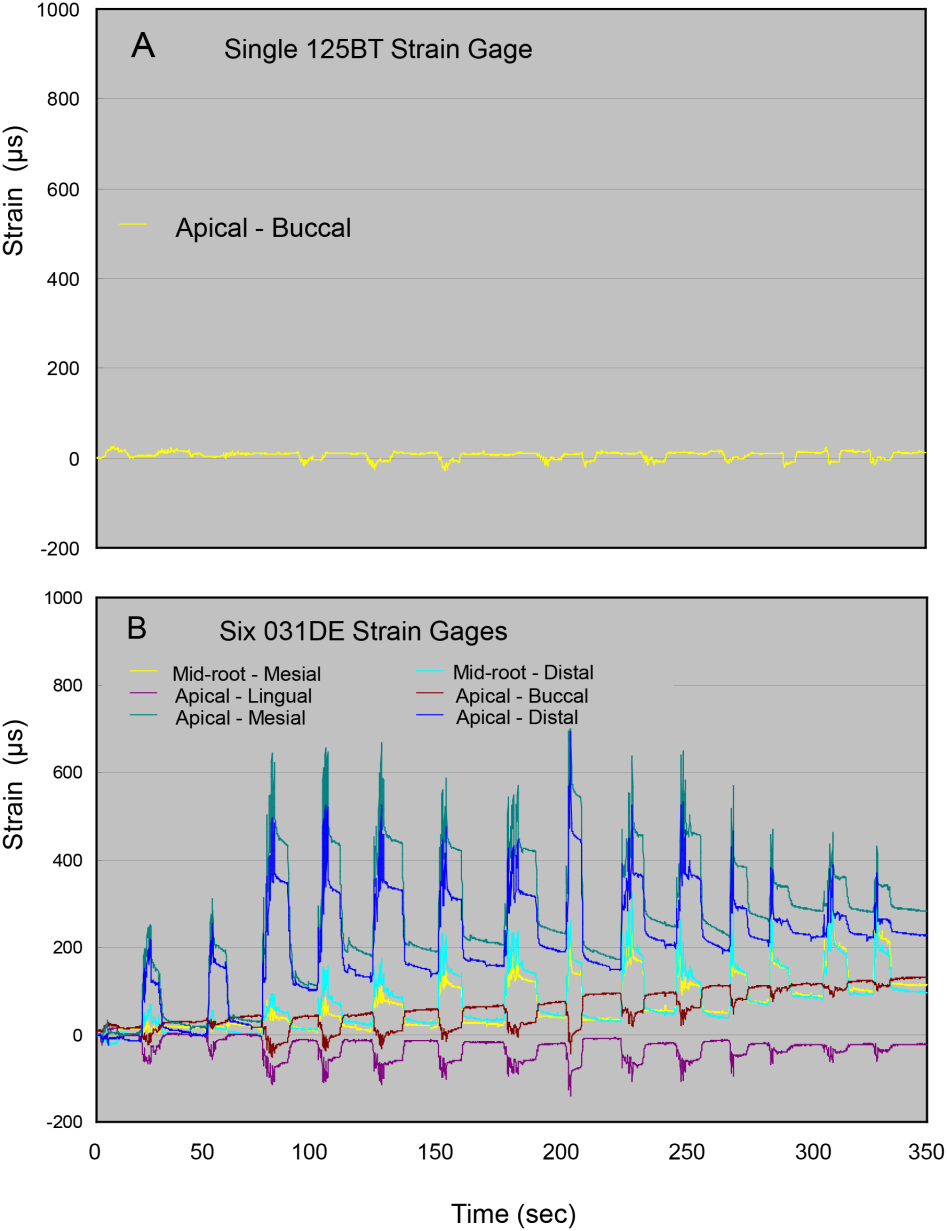
The monitored strains induced by lateral compaction in one of the control teeth. (A) A large strain gauge (125BT strain gauge) mounted on the buccal surface at the apical level of the root. (B) Six miniature strain gauges mounted on the buccal, lingual, mesial and distal aspects at the apical level of the root and on the mesial and distal aspects at the mid-root level of the same root as in A.

Root dentin surface strain plots as a function of time, which were generated during the lateral compaction procedure for one of the control teeth, are presented in Fig 2. Lateral compaction was composed of several successive single cycles of spreader insertion, each resulting in a peak of strain. The single large gauge mounted on the buccal-apical surface of the root registered very small strain values of ±20 μS (Fig 2A). However, when six miniature gauges were mounted on the ***same root***, a very different comprehensive pattern was registered (Fig 2B). The buccal maximal strain obtained from the same location as in Fig 2A increased to +125 μS. Moreover, proximal strains as high as 700 μS on both the apical mesial and distal aspects, as well as an apical lingual strain of -100 μS, were detected for the same root (Fig 2B).

**Fig 3:**
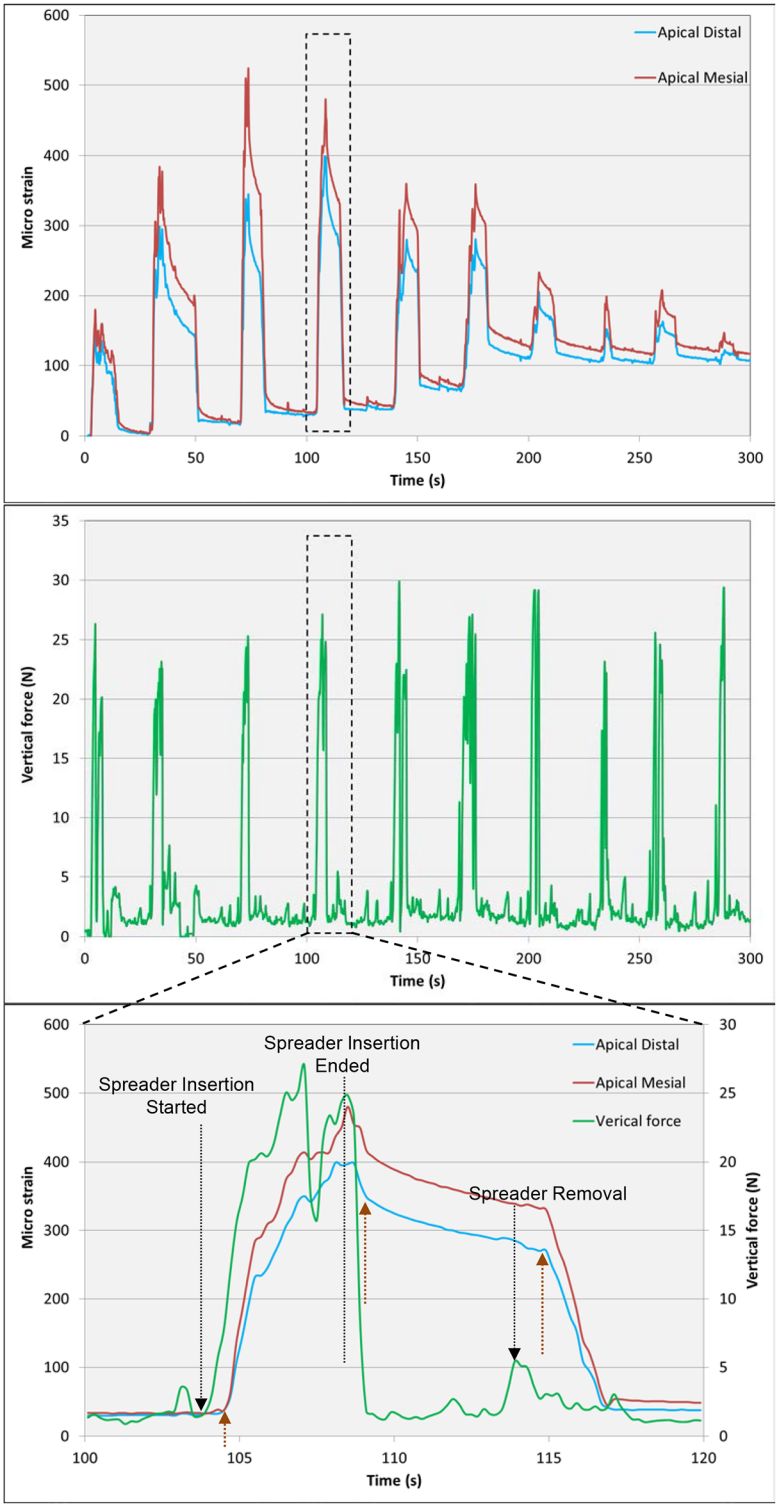
Force and strain registration during lateral compaction. (A) Strain measurements from two strain gauges located on the mesial and distal surfaces at the apical level of the root. (B) Force applied during lateral compaction. (C) The 4^th^ force and strain cycles are enlarged to facilitate detailed analysis. Note the beginning of spreader insertion, which is followed after a short lag by an increase in strain. When spreader insertion is complete and the spreader is maintained in position, no force is applied, and the strain slowly declines. Faster strain relaxation occurs after removal of the spreader, followed by the return of the strain to near background levels.

The clear and persistent finding from the output of the seven configurations assessed in this study was that the highest strains recorded during lateral compaction were in the mesial and distal aspects of the apical level of the root, and the strains decreased significantly from the apical through the mid-root to the coronal level (Fig 2B). Moreover, in all experiments, lateral compaction consisting of successive cycles of spreader insertion caused the gradual build-up of residual (offset) strains, resulting in generation of a 'stair-step' plot (Figs 2B and 3A).

Fig 3A and 3B present the apical mesial and distal strains derived from one of the teeth, which had the strain gauge configuration B (apical M+D, middle M+D, and coronal M+D) combined with relevant vertical force applied during the compaction procedure. The manner in which the spreader was used simulated the clinical lateral compaction procedure. This phenomenon is clearly reflected in the 2 diagrams shown in Fig 3A & 3B by the peaks of the force and the outcome of the strain, which are related to each spreader insertion cycle.

Measurement of the length of entry at each spreader insertion cycle from a fixed reference point and determination of the location of the tip of the spreader relative to the center of the strain gauge permitted the definition of a zone located between 2.0 mm apical and up to 1.0 mm coronal to the center of the gauge. This zone, which was termed the 'activation zone', yielded the highest strains when it was reached by the spreader during insertion (cycles 2–6 in Fig 3A). Each insertion of the spreader generated strains that followed the force, provided that the spreader tip was within the 'activation zone'. When the spreader no longer entered the 'activation zone' during subsequent insertions, the force induced minimal strains in the area of that specific gauge. However, residual strains remained.

Fig 3C shows a magnification of one compaction cycle (spreader insertion started, spreader insertion ended, spreader maintained within the gutta-percha and spreader removed). Characteristic behaviors of force and strains were observed. When the force induced by the spreader increased, the strains increased, but this occurred after a lag of approximately 0.5–0.75 s. When the spreaders were left in place while the practitioner selected an accessory cone and did not exert any manual vertical force, the strain was maintained but decreased slowly. When the spreader was removed (to allow for insertion of an accessory cone) and no force was applied, the strains quickly diminished (Fig 3C). In all experiments, residual strains persisted at the end of the procedure.

**Fig 4:**
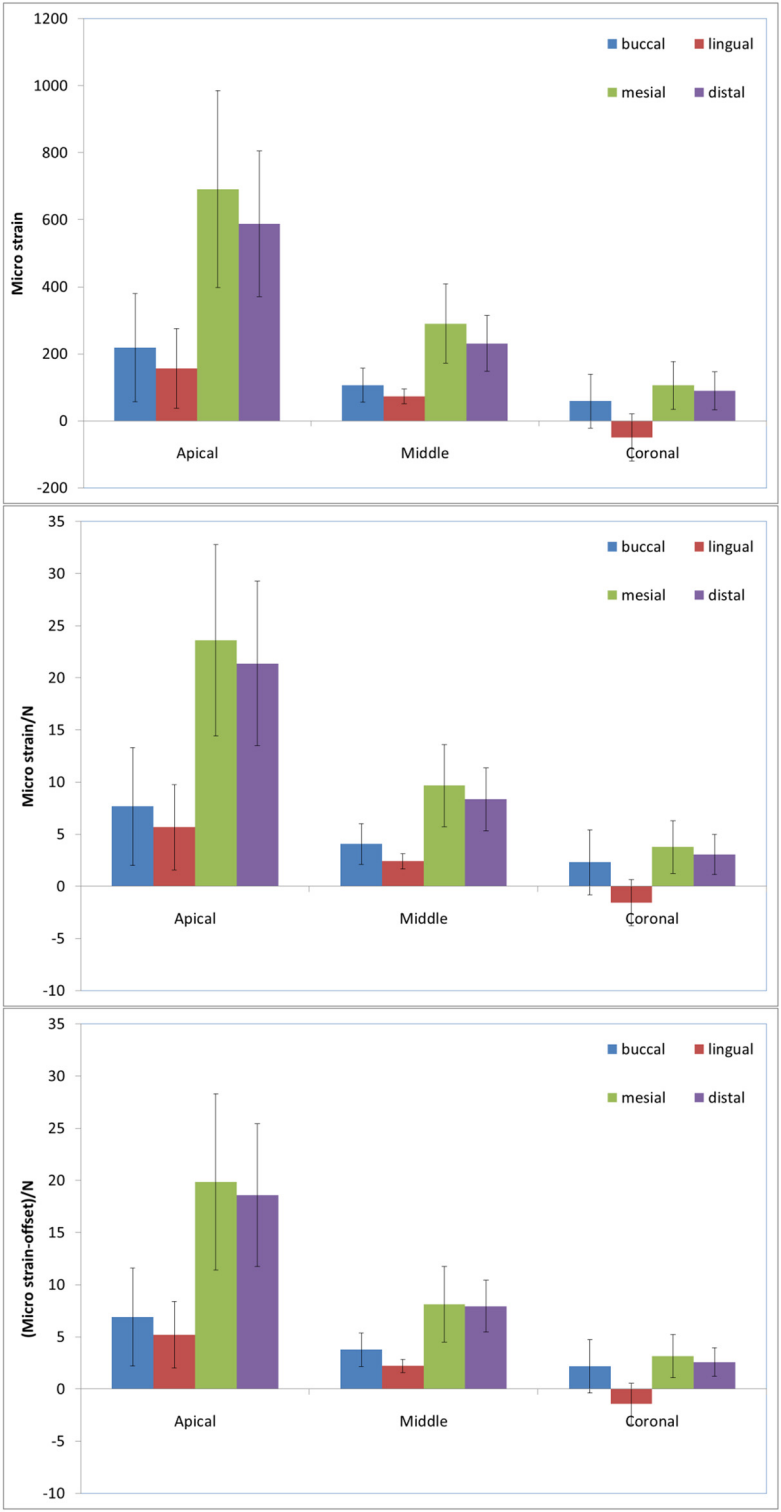
Mean maximal strains (SD) at the various aspects (buccal, lingual, mesial, and distal) and levels (apical, middle, and coronal) of the roots. (A) As measured. (B) Normalized relative to the matched force. (C) After subtracting the offset strain, normalized relative to the matched force.

From the force and strain plots, the following two parameters were further considered: the maximal strain developed for each strain gauge during the procedure and the related force. The following two variables were calculated: the maximal strain normalized relative to the matched force, and the maximal minus the offset strain normalized by the same force. These results are presented in Fig 4 for all 12 locations of strain gauges on the roots derived from the seven measurement configurations.

The magnitudes of the maximal proximal strains at the apical level as well as the mid-root were approximately 2.5–3 times higher than the buccal/lingual strains (p = 0.041) (Fig 4). There was a gradual but significant decline in both proximal and buccal/lingual strains from the apical level through the mid-root to the coronal level of the root. The apical strains were 2.5-fold higher compared with the mid-root strains (p = 0.037) and 6-fold higher compared with the strains at the coronal level (p = 0.013) (Fig 4). The mesial and distal strains did not differ significantly; however, the buccal strains were generally 35–65% higher than the lingual strains (p = 0.078). The lowest strains were recorded at the coronal level, and they were positive (tension) or negative (compression).

As previously stated, residual strains persisted at the end of each lateral compaction procedure (300 μS in Fig 2B and 110 μS in Fig 3A). Maximal residual strains of as high as 400 μS were detected by the screening of all sample sizes. These strains were later followed by continuous registration of strains at a lower frequency of 2 Hz for an additional 1000 seconds after completion of the lateral compaction. The residual strains decreased gradually. Nevertheless, small residual strains of 30 μS remained.

The vertical forces applied during the fourteen lateral compaction procedures, including all sample teeth, ranged from 12 N (usually for the earliest cycles of spreader insertion) to 36 N (usually for the maximal strain or later condensation cycles). The mean compaction force in the maximal strain cycles was 28.75±2.16 N.

## Discussion

Using multiple miniature strain gauges enabled us, for the first time, to exactly quantify the circumferential strains along the root that were induced by finger spreaders during lateral compaction. The working hypothesis was thus supported because with proper mounting of several miniature strain gauges at the various levels (apical, middle or coronal) and aspects (buccal, lingual, distal or mesial) of the roots, appreciable circumferential strains were monitored under clinically relevant compaction forces. Due to the limited availability of teeth fulfilling the inclusion criteria, not all possible combinations of the six miniature strain gauges were tested; nevertheless, the seven chosen configurations chosen enabled us to clearly differentiate the strains obtained from the various levels and aspects of the roots.

The highest strains were observed in the mesial and distal aspects of the apical level of the root, consistent with the reports of Walton et al. [37] and Saw and Messer [28], with the caveat that those studies focused on the buccal aspect alone. Saw and Messer [28] used large strain gauges with an active gauge length of 3.18 mm mounted on the buccal aspect of the root, and they reported that the strains during lateral compaction were 70% higher in the apical compared with the coronal third of the root. With six miniature 0.79-mm strain gauges bonded at three levels of the root in various configurations, we demonstrated that a larger gradient of strain existed between the apical and coronal third of the root. The apical strains were 2.5- and 6-fold higher compared with the mid-root and coronal strains, respectively.

Such differentiation between the magnitudes of strains at different levels and aspects of the root could not be achieved using strain gauges with larger dimensions, such as those used in previous studies [4, 15]. This observation was clearly demonstrated in the control teeth, for which both a single large gauge and six miniature gauges were tested on the same teeth (Fig 2). Moreover, the array of strain gauges in the different configurations allowed us to determine the locations of the highest strains along the surface of the root.

The magnitudes of the maximum proximal strains at the apical level as well as the mid-root were 2.5-3-fold higher than those at the buccal/lingual aspects. The proximal strains were consistently tensile, whereas on the buccal-lingual surfaces, they were either tensile or compressive; strains were more often compressive at the coronal level and in the lingual aspect, suggesting the involvement of bending or non-axial loading. Such behavior is attributed to the non-uniform angle of force delivery while obturation causes bending and consequently longitudinal (coronal-apical) strains. Such strains might influence the magnitude of circumferential strains due to the Poisson behavior of the dentin, which has been reported to have a value of 0.31 [12].

The tensile strains on the proximal surfaces are much more significant, not only because of their higher magnitudes but also because dentin fracture in damage-tolerant models results from tensile stresses [18]. In agreement with the current study, high tensile strains on the proximal surfaces and much lower (tensile or compressive) strains on the buccal-lingual surfaces have been reported previously by Lertchirakarn et al. [29] in a destructive model of loading to failure of the maxillary and mandibular incisors using hand spreaders. However, there are no similarities between that model and the clinical process of lateral compaction. Moreover, forces exceeding 10-fold of that which is applied in daily practice by endodontists cannot be extrapolated to clinical lateral compaction procedures.

Important variables that may account for the differences in circumferential strains between teeth include the hydration of the dentin and the dentin wall thickness. Weight loss by evaporation from human dentin at 22.3°C and 53.2% relative humidity has been reported to be rapid and non-linear, reaching 3.33 ± 0.63% after 7 days [38]. Dehydrated dentin exhibited a linear stress-strain relationship with three-point bending testing, whereas hydrated and rehydrated dentin was characterized by a lower proportional limit and greater strain [39]. Stress at fracture did not differ significantly between hydrated, dehydrated, or rehydrated dentin in bending or tensile tests, whereas strain at fracture and fracture energy were significantly greater for hydrated and rehydrated than for dehydrated dentin [39, 40]. Thus, it can be postulated that if the dentin in the current study underwent some degree of dehydration, the strain levels would have been higher in the fully hydrated state. This is the reason that careful attention was exerted to keep the dentin moist throughout the experiment. This was achieved by maintaining moisture on the inner side of the root by the continuous presence of saline in the canal, until the lateral compaction procedure was performed. To ensure that this procedure had no influence on the functioning of the strain gauges, a mounting protocol that utilized an inner thin layer of special epoxy cement (hydrophobic) under the cyanoacrylate cement was used.

Finite-element models demonstrated that the decreased radius of curvature of the inner root canal walls had a strong effect on the stress distribution due to spreader- induced stresses within the canal during the lateral compaction procedure. Uneven wall thickness in the different aspects of the root and, in particular, a reduction in the proximal wall thickness also had some influence [29, 41]. Reduction of proximal thickness compared to buccal-lingual thickness is less typical in central incisors and more typical in mandibular incisors, mesial roots of mandibular molars, and upper premolars [41].

Previous studies investigating spreader-induced root strains by axial static loads operated through universal testing machines have the benefit of highly standardized force [4, 6, 36, 42, 43]. However, FEAs have clearly demonstrated that the distribution of stress within root dentin during lateral compaction is completely dependent on model assumptions of spreader operation and procedural inconsistencies [20, 22, 44]; for example, those associated with axial rotation during translation. The model used in the present study aimed to apply finger spreaders by manual insertion with back-and-forth rotation, simulating the routine clinical procedure of lateral compaction. Therefore, to overcome the lack of standardization of force, normalization of the strains by force was needed. The mean compaction force in the maximal strain cycles was 28.75±2.16 N, which is within the acceptable 15- to 30-N mean range used in routine daily practice for root canal obturation [34–35], but forces as high as 36 N were also detected.

Normalization of strain by force, with or without subtracting the offset strain, did not change the results qualitatively but could have slightly diminished the differences. For example, the ratios between the maximal mesial variables and the buccal ones at the apical level of the root were 3.16, 3.08 and 2.87 (Fig 4) for the maximal strain, maximal strain normalized by the applied spreader load, and maximal minus offset strain normalized by the applied spreader loads, respectively.

The successive compaction cycles, allowing for the insertion of accessory cones, resulted in generation of a pseudo 'stair-step’ curve with a gradual increase in offset strain. Similar behavior to the gradual increase in offset strain during the lateral compaction procedure has been described previously by Dang and Walton [4], who postulated that these strains are ***stored*** in dentin and remain quiescent for life or until additional stresses applied through restoration or mastication convert them into complete fractures. The results of the present study reject the above assumption because the residual strains at the end of the procedure declined gradually to almost negligible levels after 1000 sec.

The strains for a specific strain gauge developed when the tip of the spreader entered the zone defined as the 'activation zone' of that strain gauge. After generalizing all of the lateral compaction procedures utilized in the present study, the 'activation zone' could be defined as the area between 2.5 mm apical and up to 1.5 mm coronal to the center of the gauge. This zone was especially pronounced at the apical level of the root. This concept supports previous studies showing that the highest strains that develop in root dentin during lateral compaction procedures are around the tip of the spreader, especially in the apical area [19, 20, 25].

Manual insertion of the spreader during lateral compaction simultaneously involved three components: translation, axial rotation and apical compaction force. The current model involved the output of all three components: the degree of translation is derived from the penetration depth measured from a predetermined fixed point, the extent of apical compaction force is derived from measurements of the load cell, and axial rotations are registered in the force and strain plots. Focusing on a single compaction cycle with numerous measurements due to a high frequency of data collection enabled us to divide the maneuver into several steps: spreader insertion initiation, spreader insertion completion, spreader maintenance within the gutta-percha and spreader removal. Thus, the detailed strain and force plots generated might serve not only as a research but also as a training tool.

Generally, the plot of the generated strains follows that of the force; however, a phase lag of 0.5–0.75 sec existed between the cause (force) and outcome (strain) at the start of force induction by the spreader, as well as upon removal of the spreader. There was a negligible phase lag between the force and the strain after completion of translation of the spreader. The apical compaction force applied adjacent to gutta-percha leads to pressure in the gutta-percha, resulting in circumferential tensile stresses on the canal surface [11]. Because the mechanical properties of gutta-percha correspond to a typical viscoelastic, partially crystalline material [45], the phase lag might be due to creep compliance of the gutta-percha, and initial deformation of the investment material can be a contributing factor. When the spreader was left in place with no application of force, the strain declined slowly, potentially due to stress relaxation of the gutta-percha.

It cannot be precluded that canal preparations with different sizes and tapers may affect strain development at the outer surface of the root. Similarly, other types of spreaders, such as stainless steel finger spreaders or hand spreaders, may also affect the strain generation. Nevertheless, such parameters were beyond the scope of the present study.

Characteristic clinical reports have provided evidence that VRFs occur more frequently in the buccal-lingual direction [15, 33, 46]; however, in the present study, maximal circumferential external strains developed on the proximal surfaces. This apparent contradiction has been previously explained by Lertchirakarn et al [29, 41] in an FEA study of VRF. The authors postulated that when the canal shape or the external root cross-sectional shape is not circular but oval, the stress distribution becomes asymmetrical, with a tendency for the highest stresses to occur in the buccal-lingual direction ***on the inner canal wall***. Fracture thus initiates from the site of greatest curvature of the root canal wall and propagates to the outer root surface. This phenomenon was more evident in the mandibular incisor, with its more pronounced oval root shape and ribbon-like canal shape [29, 41]. Chai and Tamse [17], in a 2-dimensional fracture mechanics analysis, recently approved and extended that explanation to roots with 2 canals and an isthmus. Hence, VRF is not the direct outcome of hoop stresses that are uniformly distributed around the canal but rather is a result of asymmetrical stress concentrations.

However, one must bear in mind that fracture mechanics and FEA do not take into account important parameters and make many simplifications. Ovoid canals are turned into elliptic cavities, all materials (dentin, periodontal ligament, bone, gutta-percha, and spreader) are assumed to be isotropic and linearly elastic, canal wall pressure is unrealistic, and lateral compaction is not divided into several cycles. Thus, the applicability of those models to the clinical lateral compaction procedure is questionable, and whether the outer surface root strains directly registered by the strain gauges in the current study can predict VRF merits further investigation. Future research should focus not only on the absolute values of these strains but also on changes in their patterns during the lateral compaction procedure.

## Conclusions

1. With proper mounting of several miniature strain gauges at the various levels and aspects of the root, appreciable circumferential strains can be monitored under clinically relevant compaction forces.
2. The highest strains develop in the mesial and distal aspects at the apical level of the root. These strains are consistently tensile.
3. There is a six-fold gradient of strain between the apical and coronal levels of the root.
4. The successive compaction cycles allowing for the insertion of accessory cones results in generation of a pseudo stair-step curve with a gradual increase in offset strain.
5. The residual strains at the end of lateral compaction are not stored in dentin but decline gradually and almost completely after 1000 sec.
